# The association between physical activity and healthcare costs in children – results from the GINIplus and LISAplus cohort studies

**DOI:** 10.1186/s12889-015-1721-6

**Published:** 2015-04-29

**Authors:** Nadja Idler, Christina M Teuner, Matthias Hunger, Rolf Holle, Sandra Ortlieb, Holger Schulz, Carl-Peter Bauer, Ute Hoffmann, Sibylle Koletzko, Irina Lehmann, Andrea von Berg, Dietrich Berdel, Barbara Hoffmann, Beate Schaaf, Joachim Heinrich, Silke B Wolfenstetter, for the GINIplus and LISAplus Study Groups

**Affiliations:** Helmholtz Zentrum München, German Research Center for Environmental Health, Institute of Health Economics and Health Care Management, Neuherberg, Germany; Ludwig-Maximilians-Universität München, Munich School of Management – Institute of Health Economics and Health Care Management and Munich Center of Health Sciences, Munich, Germany; Institute of Epidemiology I, Helmholtz Zentrum München, German Research Center for Environmental Health, Neuherberg, Germany; Technical University of Munich (TUM), Institute for Medicinal Statistics and Epidemiology, Munich, Germany; Department for Paediatrics, Technical University of Munich (TUM), Munich, Germany; Ludwig-Maximilians-Universität München, Dr. von Haunersches Children’s Hospital, Munich, Germany; Department of Environmental Immunology, Helmholtz Centre for Environmental Research Leipzig (UFZ), Leipzig, Germany; Department of Paediatrics, Marien-Hospital Wesel, Wesel, Germany; University of Düsseldorf (IUF), Leibniz Research Institute for Environmental Medicine and Medical Faculty, Düsseldorf, Germany; Medical Practice for Paediatrics, Bad Honnef, Germany

**Keywords:** Physical activity, Healthcare utilization, Healthcare costs, Direct costs, Indirect costs, Children, Cross-sectional study

## Abstract

**Background:**

Physical inactivity in children is an important risk factor for the development of various morbidities and mortality in adulthood, physical activity already has preventive effects during childhood. The objective of this study is to estimate the association between physical activity, healthcare utilization and costs in children.

**Methods:**

Cross-sectional data of 3356 children aged 9 to 12 years were taken from the 10-year follow-up of the birth cohort studies GINIplus and LISAplus, including information on healthcare utilization and physical activity given by parents via self-administered questionnaires. Using a bottom-up approach, direct costs due to healthcare utilization and indirect costs resulting from parental work absence were estimated for the base year 2007. A two-step regression model compared effects on healthcare utilization and costs for a higher (≥7 h/week) versus a lower (<7 h/week) level of moderate-to-vigorous physical activity (MVPA) adjusted for age, gender, BMI, education and income of parents, single parenthood and study region. Recycled predictions estimated adjusted mean costs per child and activity group.

**Results:**

The analyses for the association between physical activity, healthcare utilization and costs showed no statistically significant results. Different directions of estimates were noticeable throughout cost components in the first step as well as the second step of the regression model. For higher MVPA (≥7 h/week) compared with lower MVPA (<7 h/week) total direct costs accounted for 392 EUR (95% CI: 342–449 EUR) versus 398 EUR (95% CI: 309–480 EUR) and indirect costs accounted for 138 EUR (95% CI: 124–153 EUR) versus 127 EUR (95% CI: 111–146 EUR).

**Conclusions:**

The results indicate that childhood might be too early in life, to detect significant preventive effects of physical activity on healthcare utilization and costs, as diseases attributable to lacking physical activity might first occur later in life. This underpins the importance of clarifying the long-term effects of physical activity as it may strengthen the promotion of physical activity in children from a health economic perspective.

## Background

Physical activity (PA) has several preventive effects on physical and mental health [1-3]. Physical inactivity has been labeled as a pandemic and is the fourth leading risk factor for global mortality [4,5]. It is estimated that worldwide about 3.2 million deaths and 32.1 million disability-adjusted life years (DALYs) are annually attributable to insufficient PA [6]. Physical inactivity accounts for between 1.0 and 2.6% of the total healthcare costs in developed countries [7].

Data from the German Interview and Examination Survey for Children and Adolescents (KiGGS) show a pattern of insufficient PA for children in Germany: only 15.3% of children and adolescents between 4 and 17 years of age fulfill World Health Organization (WHO) recommendations regarding PA (moderate to vigorous intensity) of at least 60 minutes a day [5,8].

A lack of PA influences the development of obesity and a growing prevalence of obesity can be observed in children [9,10]. In Germany, 15% of the children and adolescents aged 3 to 17 years are overweight (including obesity) and 6.3% are obese [11]. Three German studies indicate that childhood obesity is a cost driver for the healthcare system [12-14].

While there has been some research on the impact of childhood obesity on healthcare utilization and costs, the association between PA, healthcare utilization and costs has barely been explored for children. A prospective Dutch study with 996 primary schoolchildren describes the economic burden of injuries that occur during physical education class, leisure time or organized sports [15]. Only one cross-sectional Canadian study analyzes the association between health behavior and healthcare utilization costs in 4380 grade 5 students from elementary schools [16]. In that study, Kirk et al. link survey data from the 2003 Children’s Lifestyle and School Performance Study (CLASS) including children’s PA and screen time with administrative health data from the province Nova Scotia (number of physician visits and physician costs for each child from 2001 to 2006) [16]. Kirk et al. find no statistically significant relationship between PA or screen time and healthcare utilization or costs. A non-significant trend shows increasing healthcare costs for increasing PA and decreasing screen time [16].

Until now, there are no studies in Germany analyzing the relationship between PA, healthcare utilization and costs for children. Assuming preventive effects of PA on healthcare utilization and costs on the one hand and economic burden resulting from PA-related injuries on the other, it is still uncertain whether savings or additional costs predominate in physically active children. The aim of this cross-sectional study is to analyze the correlation between different levels of PA and healthcare utilization as well as costs for children aged 9 to 12 years based on data from the GINIplus- and LISAplus studies.

## Methods

### Study population and sampling

The cross-sectional data were taken from the 10-year follow-up of two prospective population-based birth cohort studies: The GINIplus study (The German Infant Study on the Influence of Nutrition Intervention plus Air Pollution and Genetics on Allergy Development) and the LISAplus study (Influence of Life-style Factors on Development of the Immune System and Allergies in East and West Germany plus Air Pollution and Genetics on Allergy Development). Included in both studies were healthy, fullterm newborns with a birth weight >2500 g who are of German descent and live in the proximity of study centers in Munich, Leipzig, Bad Honnef and Wesel. In these areas, newborns were recruited from obstetric clinics between the mid- to the late 1990s [17]. Further inclusion criteria, the intervals analyzed, the design of the study arms and the interventions are described in more detail elsewhere [17,18].

Both study protocols were approved by the local ethic committees (Bavarian General Medical Council, University of Leipzig, Medical Council of North-Rhine-Westphalia) and written informed consent was obtained from all participating families.

Starting with 5991 newborns in GINIplus and 3097 newborns in LISAplus at baseline, after 10 years about 55% of all individuals were left for data collection. The cross-sectional data for the 10-year follow-up (mean age of individuals: 10.08 years) were available between 2005 and 2009 depending on the birth date of the individuals [17]. The 10-year follow-up of both studies provides data for 5049 children [14]. For the first time, a questionnaire recording healthcare utilization was applied, resulting in data for 3642 children. The aim of applying this questionnaire was to analyze the costs resulting from healthcare utilization in children [17].

Data on the exposure PA are missing for 286 of those children. To avoid unnecessary further loss of data, we included children in the analyses for whom only data on covariates were missing but data for the exposure PA were available. Thus, the analyses are based on data for 3356 individuals.

### Definition of physical activity

PA is a generic term for any movement of the body which is produced by the skeletal muscles and increases energy use above the metabolic rate at rest [19]. In this study, using a self-administered questionnaire, parents of participating children had to assess the intensity and a mixed dimension of quantity and frequency of their children’s PA. Possible response categories of intensity were “light PA” (without sweating, normal respiration, e.g. walking), “moderate PA” (some sweating, slightly increased breathing, e.g. cycling, swimming, skating) and “vigorous PA” (a lot of sweating and fast breathing, e.g. ball games, training). For each intensity category, parents were asked to estimate the mixed dimension of quantity and frequency of their children’s PA in hours per week (h/week) separately for summer and for winter time. Mean annual values were calculated for each child in each intensity category.

The WHO and various other guidelines advise children to be physically active on a moderate-to-vigorous intensity to maintain a basic level of health [5,20,21]. WHO-guidelines for children recommend 60 minutes moderate-to-vigorous PA (MVPA) per day [5].

Therefore, hours of moderate PA and vigorous PA that were reported by the parents of participating children were added up to build a sum variable of MVPA weighting moderate and vigorous PA equally and combining the information on intensity, quantity and frequency of PA in h/week MVPA.

For the primary analysis, the sum variable MVPA was dichotomized into two levels : <7 h/week MVPA (WHO-recommendations not met) and ≥7 h/week MVPA (WHO-recommendations met). A four-level MVPA-variable was considered in a sensitivity analysis model categorized in: <3.5 h/week MVPA, ≥3.5 h/week and <7 h/week MVPA, ≥7 h/week and <10.5 h/week MVPA, ≥10.5 h/week MVPA.

### Socioeconomic factors and BMI

Covariates used in the analysis are age, gender, body mass index (BMI) of children, highest education level of parents, relative income position of the household (relative to the median equivalence income), single parenthood and study region. Socioeconomic information was obtained from parents via self-administered questionnaires.

As measures of children’s socio-economic background information on the education level and income of parents were used. Education level of parents was given by the maximum completed school years of either of the parents: “low” (<10 years), “medium” (=10 years), “high” (>10 years). In cases of missing information (0.4% of mothers, 2% of fathers) single imputation was conducted, applying the Markov Chain Monte Carlo method (PROC MI in SAS) [22]. Completed education levels served to impute missing income positions (9.8% of cases) using the logistic regression method within PROC MI [22].

Information on parents’ net household income was converted into equivalence income according to the modified Organization for Economic Cooperation and Development (OECD) scale [23,24]. Equivalence income considers the size of the household and weights its members to reflect the households' spending capacity more precisely. According to the EU convention, the threshold value of poverty risk is defined as 60% of the median net equivalence income [25]. Using the median equivalence income of Germany in 2007 as a reference (1521 EUR/month)^a^ [23], the relative income position of the household was categorized into: ≤ 60% of median equivalence income, > 60 and ≤ 100% of median equivalence income and > 100% of median equivalence income.

Anthropometric data of weight and height were recorded at the physical examination of the children by trained medical staff and BMI was calculated as weight in kilograms divided by height in meters squared. BMI data were classified according to German age- and sex-specific percentile cut-off points for children, resulting in the categories “severely underweight” (<P3), “underweight” (P3 to < P10), “normal weight” (P10 to P90), “overweight but not obese” (>P90 to P97) and “obese” (>P97) [26,27].

### Healthcare utilization, direct and indirect costs

In the healthcare utilization questionnaire parents reported whether their child had used healthcare services (physician, therapist, hospital, rehabilitation) as well as the number of physician visits (pediatrician, general practitioner, ophthalmologist, orthopaedist, ear, nose and throat specialist (ENT), dermatologist, pulmonologist, emergency doctor and other specialist), therapist visits (alternative practitioner, physiotherapist, speech therapist, psychotherapist, occupational therapist, homeopath, other therapist), the number of hospital days and inpatient rehabilitation days of their children. In addition parents provided information on their work absence days (occurred yes/no, number of days) required due to health problems of their children. All questions referred to the previous 12 months.

On the basis of this individual level data direct medical costs were assessed applying a bottom-up approach and unit prices. Physician costs were calculated using prices per physician visit for each medical specialty, taken from a national costing guideline from the Working Group Methods in Health Economic Evaluation (AG MEG) [14,28]. For the estimation of therapist costs the number of visits were multiplied by the appropriate valuation rate suggested by the AG MEG and supplemented by information from relevant organizations [14,29]. The costs of hospital visits and inpatient rehabilitation stays were assessed by multiplying the number of days by the mean costs per day [14]. Utilization and costs of pharmaceuticals were not included in this analysis.

Direct medical costs were calculated as total costs (sum of physician, therapist, hospital and inpatient rehabilitation costs) as well as in the separate subcategories: physician, therapist, hospital and inpatient rehabilitation costs.

Indirect costs resulting from parental work absence were calculated as age- and gender-specific mean costs per day of lost work for employees multiplied by the number of lost work days caused by the child [14]. The human capital approach was applied to evaluate production losses [14,30].

All costs were denominated in Euros and referred to the year 2007 [14]. More details on monetary valuation and imputation procedures can be found elsewhere [14,22]. For sensitivity analyses of costs to changes in the assumptions regarding valuation methods and imputation procedures, see Breitfelder et al. [14]. For all cost components an excess cost approach was applied, i.e. all costs due to both the exposure variable MVPA and consequences of MVPA on health were captured [13,31]. This allowed for comparison of MVPA groups regarding cost differences.

### Statistical analysis

Differences of variable distributions for both cohorts (GINIplus and LISAplus) have already been analyzed elsewhere and no noticeable differences were found [32]. Therefore, the data from both studies were pooled together. Descriptive analyses provide an overview of the study population including PA behavior of the children and their utilization of healthcare services. For this purpose, absolute frequencies (plus percentage values) and mean values (plus standard deviation) were calculated.

Bivariate analyses were conducted for the MVPA variable of primary analysis and each covariate separately, for the variables education level and relative income position as well as for the MVPA variable and each cost category.

The primary regression model analyzes the association between MVPA and direct medical costs (total costs, physician costs, therapist costs, hospital costs and inpatient rehabilitation costs) as well as the association between MVPA and indirect costs (costs of parental work absence). The regression model was adjusted for the covariates age, gender, BMI of children, education level of parents, relative income position of the household (relative to median equivalence income), single parenthood and study region.

Descriptive analysis of the outcome total costs showed a positively skewed distribution. To account for positively skewed cost data and for a high number of zero-costs (13.4% of total costs), a two-step regression approach was applied to model the costs. The first step consists of a logistic regression model (PROC LOGISTIC in SAS). Handling a binary response variable regarding utilization (yes/no) it estimates the association between MVPA, covariates and the odds of generating costs in a particular cost category. In the second step, a generalized linear regression model (PROC GENMOD in SAS) was used to assess the association between MVPA, covariates and the extent of costs caused by the healthcare utilization of the children in a particular cost category. A gamma distribution with log-link function was assumed [33].

Four types of sensitivity analyses were conducted on each of the cost components and for both steps of the model. Sensitivity analysis model 1 (SAM 1) reperformed the primary analysis excluding two individuals due to high utilization of healthcare services (>100 days of stay in hospital or inpatient rehabilitation). In SAM 2 the categorization of the MVPA variable was changed from dichotomous into four-level: <3.5 h/week MVPA, ≥3.5 and <7 h/week MVPA, ≥7 and <10.5 h/week MVPA and ≥10.5 h/week MVPA. Two further models were adjusted for the same covariates as the primary analysis, but in each instance one extra variable was included in the calculation: SAM 3) an interaction term of MVPA and gender, SAM 4) an interaction term of MVPA and BMI. All sensitivity analyses were considered as explorative, therefore no adjustment for multiple testing was made.

Additionally, recycled predictions were applied combining both steps of the regression model to assess the overall association between PA and costs. Therefore, adjusted mean costs and the cost differences were estimated for the two MVPA groups (WHO recommendations met vs. WHO recommendations not met). 1000 bootstrap replications and the percentile method were used to estimate 95%-bootstrap-percentile-intervals [34,35]. For statistical calculations the software package SAS (SAS Institute Inc., Cary, NC, USA, Version 9.2) was used and p-values ≤ 5% were considered statistically significant.

## Results

### Description of the study population and healthcare utilization

Table 1 shows the characteristics of the study population in absolute and relative frequencies or mean values plus the standard deviation. Participating children are quite active, the majority fulfill WHO recommendations: for 69.3% of all children, parents reported ≥ 7 h/week MVPA. The four-level MVPA variable reveals that 44.0% even do more than 10.5 h/week MVPA.

**Table 1:** **Description of the study population (GINIplus and LISAplus)**

**Subject characteristic**	**Analyzed population (N = 3356)**		**Population according to MVPA activity time** ^**4**^		**Bivariate analysis**
	**N**	**Mean (SD) or %**	**Mean (SD) or N (%) doing**	**Mean (SD) or N (%) doing**	**MVPA (h/week) and** ^**7**^
			**<7 h/week MVPA** ^**5**^	**≥7 h/week MVPA** ^**6**^	
			**(N = 1030)**	**(N = 2326)**	
**Age**	3356	10.08 (0.23)	10.09 (0.25)	10.08 (0.22)	t = 0.93, p = 0.35 (TT)
**Gender**					*χ* ^2^ = 0.78, p = 0.38 (PT)
Boys	1723	51.34	517 (50.19)	1206 (51.85)	
Girls	1633	48.66	513 (49.81)	1120 (48.15)	
**BMI categories** ^**1**^					*χ* ^2^ = 10.22, p = 0.07 (PT)
Severely underweight (< P3)	89	2.65	22 (2.14)	67 (2.88)	
Underweight (P3 to < P10)	212	6.32	60 (5.83)	152 (6.53)	
Normal weight (P10 to P90)	2674	79.68	850 (82.52)	1824 (78.42)	
Overweight, not obese (> P90 to P97)	194	5.78	45 (4.37)	149 (6.41)	
Obese (> P97)	60	1.79	20 (1.94)	40 (1.72)	
Missing	127	3.78	33 (3.20)	94 (4.04)	
**Education level of parents** ^**2**^					*χ* ^2^ = 0.08, p = 0.96 (PT)
Low (<10 years)	174	5.18	52 (5.05)	122 (5.25)	
Medium (=10 years)	897	26.73	274 (26.60)	623 (26.78)	
High (>10 years)	2285	68.09	704 (68.35)	1581 (67.97)	
**Relative income position** ^**3**^					*χ* ^2^ = 2.80, p = 0.25 (PT)
≤60% of median income	539	16.06	151 (14.66)	388 (16.68)	
>60 and ≤ 100% of median income	1191	35.49	362 (35.15)	829 (35.64)	
>100% of median income	1626	48.45	517 (50.19)	1109 (47.68)	
**Single parenthood**					*χ* ^2^ = 10.34, p = 0.01 (PT)
Yes	334	9.95	124 (12.04)	210 (9.03)	
No	2965	88.35	895 (86.89)	2070 (88.99)	
Missing	57	1.70	11 (1.07)	46 (1.98)	
**Study region**					*χ* ^2^ = 24.68, p < 0.0001 (PT)
Munich	1717	51.16	551 (53.49)	1166 (50.13)	
Leipzig	354	10.55	132 (12.82)	222 (9.54)	
Bad Honnef	185	5.51	66 (6.41)	119 (5.12)	
Wesel	1100	32.78	281 (27.28)	819 (35.21)	

Abbreviations: BMI: body mass index; h: hours; N: number of observations; P: percentile; %: percentage; PT: Pearson chi-square test; SD: standard deviation.

TT: *t*-test for independent samples.

^1^BMI categories according to Kromeyer-Hauschild et al. 2001 [28].

^2^Maximum completed school years of either of the parents; ^3^categorized relatively to the median equivalence income of the year 2007 (1521 EUR/month) [24].

^4^MVPA: moderate-to-vigorous physical activity (year average, dichotomized according to WHO recommendations ≥ 60 min/day MVPA) [2].

^5^WHO recommendations not met; ^6^WHO recommendations met.

^7^Bivariate analysis of the variable MVPA with each single covariate (age, gender, BMI, education, income, single parenthood, study region).

Bivariate analyses for the dichotomous MVPA variable and covariates show that there is no significant association between MVPA and gender, MVPA and BMI, MVPA and education level of parents or MVPA and relative income position of the household (Pearson chi-square tests). Bivariate analysis for MVPA and age was conducted using the *t*-test for independent samples. There is no significant difference in mean values for age between MVPA groups. Significant associations were observed for MVPA and single parenthood as well as for MVPA and study region (Pearson chi-square tests).

In Table 2, an overview of healthcare utilization and parental work absence is provided. The calculation shows absolute and relative frequencies of subjects using resources plus their mean frequencies of utilization (visits or days of stay) over the past 12 months. Standard deviations of the mean frequencies of utilization are high.

**Table 2:** **Utilization of healthcare services and parental work absence**

**Healthcare service components**	**Analyzed population (N = 3356)**				**Bivariate analysis**
	**Subjects using resources**		**Mean frequency of utilization (if used)**	**SD**	**MVPA (h/week) and costs** ^**1**^
	**N**	**%**	**N**		
**Physician visits (total)**	2849	84.89	4.36	4.13	z = 0.04, p = 0.97 (WT)
Pediatrician	1885	56.17	2.60	2.45	
General practitioner	857	25.54	2.23	1.61	
Ophthalmologist	997	29.71	1.36	0.9	
Orthopaedist	501	14.93	1.69	1.24	
Ear, nose and throat specialist (ENT)	389	11.59	1.94	1.73	
Dermatologist	406	12.10	1.98	2.19	
Pulmonologist	99	2.95	2.86	3.47	
Other specialist	337	10.04	2.99	4.38	
Emergency doctor	450	13.41	1.25	0.69	
**Therapist visits (total)**	842	25.09	13.95	20.35	z = −0.12, p = 0.90 (WT)
Alternative practitioner	204	6.08	4.10	10.81	
Physiotherapist	182	5.42	14.68	16.94	
Speech therapist	132	3.93	16.59	13.92	
Psychotherapist	181	5.39	12.59	15.34	
Occupational therapist	94	2.80	21.00	16.95	
Homeopath	194	5.78	2.94	2.56	
Other therapist	97	2.89	12.64	17.91	
**Hospital (days)**	181	5.39	6.15	14.97	z = −0.89, p = 0.37 (WT)
**Inpatient rehabilitation (days)**	47	1.40	22.09	26.59	z = 0.18, p = 0.86 (WT)
**Parental work absence (days)**	836	32.84*	3.89	4.14	z = 1.11, p = 0.27 (WT)

Abbreviations: h: hours; N: number of observations; %: percentage; SD: standard deviation; WT: Wilcoxon-Mann-Whitney test; *of those who are employed: N = 2744 (not employed: N = 876).

^1^Bivariate analysis of the variable MVPA and the costs of each healthcare service component.

Results for Wilcoxon-Mann-Whitney test of MVPA and total direct costs: z = −0,25, p = 0.80.

For bivariate analysis of MPVA and cost data the non-parametric Wilcoxon-Mann-Whitney test was applied. There is no significant difference in mean costs between MVPA groups for total direct costs, physician costs, therapist costs, hospital costs, inpatient rehabilitation costs or parental work absence costs.

### Association between socioeconomic factors, BMI, MVPA and (in)direct costs

Table 3 shows the results for the first step of the regression model (logistic regression).

**Table 3:** **Association between socioeconomic factors, BMI, MVPA and (in)direct costs (logistic regression model)**

**Parameter**	**Direct costs (no/yes)**					**Indirect costs (no/yes)**
	**Total**	**Physician**	**Therapist**	**Hospital**	**Inpatient rehabilitation**	**Parental work absence**
	**Odds [95% CI]**	**Odds [95% CI]**	**Odds [95% CI]**	**Odds [95% CI]**	**Odds [95% CI]**	**Odds [95% CI]**
**Age**	0.98 [0.63-1.51]	0.98 [0.65-1.48]	0.93 [0.65-1.31]	2.22 [1.28-3.86]**	1.87 [0.65-5.42]	0.86 [0.59-1.25]
**Gender, Ref: Girls**	1.00	1.00	1.00	1.00	1.00	1.00
Boys	1.17 [0.95-1.43]	1.10 [0.91-1.33]	1.21 [1.04-1.42]*	0.94 [0.69-1.27]	1.10 [0.61-1.98]	1.03 [0.87-1.23]
**BMI categories** ^**1**^ **, Ref: Normal weight (P10 to P90)**	1.00	1.00	1.00	1.00	1.00	1.00
Severely underweight (< P3)	1.81 [0.83-3.96]	1.79 [0.86-3.74]	1.14 [0.71-1.85]	1.99 [0.94-4.23]	4.23 [1.43-12.52]**	1.14 [0.66-1.98]
Underweight (P3 to < P10)	1.07 [0.70-1.63]	0.97 [0.66-1.44]	1.34 [0.98-1.82]	1.52 [0.87-2.66]	0.83 [0.20-3.50]	1.13 [0.80-1.60]
Overweight, not obese (> P90 to P97)	1.12 [0.72-1.74]	0.98 [0.66-1.47]	1.55 [1.12-2.14]**	1.49 [0.84-2.66]	0.81 [0.19-3.43]	0.93 [0.63-1.38]
Obese (> P97)	2.24 [0.80-6.26]	2.56 [0.92-7.12]	1.11 [0.61-2.03]	1.62 [0.63-4.19]	5.42 [1.96-15.03]***	1.41 [0.75-2.63]
Missing	0.67 [0.42-1.06]	0.66 [0.42-1.03]	0.64 [0.40-1.04]	1.24 [0.59-2.60]	1.72 [0.51-5.80]	1.10 [0.70-1.75]
**Education level of parents** ^**2**^ **, Ref: High (>10 years)**	1.00	1.00	1.00	1.00	1.00	1.00
Low (<10 years)	1.33 [0.81-2.19]	1.41 [0.87-2.28]	0.78 [0.51-1.19]	1.66 [0.88-3.12]	2.37 [0.84-6.67]	0.69 [0.42-1.15]
Medium (=10 years)	1.04 [0.81-1.33]	1.08 [0.85-1.37]	1.07 [0.87-1.30]	1.08 [0.74-1.57]	1.51 [0.75-3.02]	0.98 [0.79-1.22]
**Relative income position** ^**3**^ **, Ref: > 100% of median income**	1.00	1.00	1.00	1.00	1.00	1.00
≤60% of median income	0.92 [0.66-1.28]	0.89 [0.66-1.22]	1.01 [0.78-1.32]	1.15 [0.73-1.83]	1.77 [0.75-4.14]	0.77 [0.57-1.04]
>60% and ≤ 100% of median income	0.77 [0.60-0.97]*	0.81 [0.65-1.02]	1.21 [1.00-1.46]*	0.81 [0.56-1.17]	1.03 [0.48-2.23]	0.92 [0.76-1.13]
**Single parenthood, Ref: No**	1.00	1.00	1.00	1.00	1.00	1.00
Yes	0.93 [0.66-1.30]	1.00 [0.73-1.39]	1.11 [0.86-1.44]	0.75 [0.43-1.29]	1.60 [0.69-3.68]	0.81 [0.60-1.09]
Missing	0.95 [0.44-2.02]	0.84 [0.42-1.68]	1.13 [0.62-2.07]	0.60 [0.14-2.49]	<0.001 [<0.001- > 999.999]	1.57 [0.83-2.94]
**Study region, Ref: Munich**	1.00	1.00	1.00	1.00	1.00	1.00
Leipzig	1.55 [1.03-2.33]*	1.78 [1.20-2.64]**	0.82 [0.63-1.07]	1.73 [1.10-2.73]*	3.18 [1.36-7.40]**	1.97 [1.52-2.54]***
Bad Honnef	1.15 [0.70-1.88]	1.22 [0.77-1.94]	0.69 [0.48-0.99]*	1.10 [0.56-2.19]	1.09 [0.24-4.96]	0.76 [0.51-1.13]
Wesel	0.68 [0.53-0.86]***	0.75 [0.60-0.94]**	0.50 [0.41-0.61]***	0.89 [0.61-1.31]	1.53 [0.72-3.28]	0.38 [0.30-0.48]***
**MVPA activity time** ^**4**^ **, Ref: < 7 h/week MVPA** ^**5**^	1.00	1.00	1.00	1.00	1.00	1.00
≥7 h/week MVPA^6^	0.95 [0.76-1.19]	1.06 [0.86-1.30]	1.05 [0.88-1.25]	1.20 [0.85-1.68]	0.97 [0.52-1.84]	1.04 [0.87-1.26]

Abbreviations: BMI: body mass index; CI: confidence interval; h: hours, Odds: odds ratio estimates; %: percentage; Ref: reference category.

^1^BMI categories according to Kromeyer-Hauschild et al. 2001 [28].

^2^Maximum completed school years of either of the parents.

^3^Categorized relatively to the median equivalence income of the year 2007 (1521 EUR/month) [24].

^4^MVPA: moderate-to-vigorous physical activity (year average, dichotomized according to WHO recommendations ≥ 60 min/day MVPA) [2].

^5^WHO recommendations not met.

^6^WHO recommendations met.

***/**/*, values nominally significant at the 0.1%/1%/5% level (without adjustment for multiple testing); number of observations: 3356.

Model information: dependent variables: odds of direct costs (total, physician use, therapist use, hospital use, inpatient rehabilitation use) and odds of indirect costs (parental work absence);

assumptions: binominal distribution of the error terms, logit-link function.

The analysis showed no statistically significant results. Comparing MVPA ≥ 7 h/week and MVPA < 7 h/week, the odds ratio estimates (odds of generating costs, yes/no) showed different directions throughout cost components: the probabilities of total direct costs and inpatient rehabilitation costs were 0.95fold (95% CI: 0.76-1.19) and 0.97fold (95% CI: 0.52-1.84), while probabilities of physician, therapist, hospital or indirect costs were 1.06fold (95% CI: 0.86-1.30), 1.05fold (95% CI: 0.88-1.25), 1.20fold (95% CI: 0.85-1.68) and 1.04fold (95% CI: 0.87-1.26).

Regarding further parameters of the regression model, there are single significant odds ratio estimates: A one unit increase in age is associated with a twofold higher probability of hospital costs. For boys, the odds ratio estimate for therapist costs is higher compared with girls. Regarding BMI, the probabilities of inpatient rehabilitation costs for severely underweight and for obese children are higher compared with normal weight children (4.23fold, 95% CI: 1.43-12.52 and 5.42fold, 95% CI: 1.96-15.03).

More detailed analyses were done regarding the probability of costs for further specialists as well as for further therapists. The analyses did not reveal systematic changes in comparison with the reported overall analysis on the probability of physician and therapist costs.

The results for the second step of the regression model (generalized linear regression) are presented in Table 4.

**Table 4:** **Association between socioeconomic factors, BMI, MVPA and (in)direct costs (generalized linear mixed model)**

**Parameter**	**Direct costs**					**Indirect costs**
	**Total**	**Physician**	**Therapist**	**Hospital**	**Inpatient rehabilitation**	**Parental work absence**
	**Exp(Est) [95% CI]**	**Exp(Est) [95% CI]**	**Exp(Est) [95% CI]**	**Exp(Est) [95% CI]**	**Exp(Est) [95% CI]**	**Exp(Est) [95% CI]**
**Age**	1.63 [1.28-2.08]***	0.99 [0.87-1.13]	0.73 [0.51-1.04]	1.46 [0.86-2.48]	2.62 [0.75-9.16]	1.03 [0.83-1.29]
**Gender, Ref: Girls**	1.00	1.00	1.00	1.00	1.00	1.00
Boys	1.13 [1.01-1.27]*	1.06 [0.99-1.13]	1.23 [1.04-1.45]*	1.02 [0.76-1.36]	1.31 [0.64-2.70]	1.06 [0.96-1.17]
**BMI categories** ^**1**^ **, Ref: Normal weight (P10 to P90)**	1.00	1.00	1.00	1.00	1.00	1.00
Severely underweight (< P3)	1.59 [1.13-2.24]**	1.14 [0.94-1.37]	0.84 [0.51-1.36]	1.14 [0.57-2.26]	0.56 [0.08-3.68]	1.33 [0.95-1.85]
Underweight (P3 to < P10)	1.62 [1.29-2.05]***	1.16 [1.03-1.32]*	1.06 [0.76-1.46]	1.64 [1.00-2.67]*	0.48 [0.04-5.25]	1.15 [0.94-1.40]
Overweight, not obese (> P90 to P97)	1.42 [1.12-1.80]**	1.31 [1.14-1.49]***	0.85 [0.612-1.17]	1.55 [0.94-2.55]	1.16 [0.25-5.48]	1.19 [0.93-1.50]
Obese (> P97)	1.89 [1.27-2.82]**	1.16 [0.93-1.45]	1.20 [0.64-2.23]	1.29 [0.58-2.90]*	1.06 [0.37-3.08]	1.18 [0.83-1.69]
Missing	1.55 [1.14-2.12]**	0.88 [0.74-1.04]	0.70 [0.42-1.19]	2.34 [1.16-4.74]	0.37 [0.08-1.75]	0.86 [0.66-1.12]
**Education level of parents** ^**2**^ **, Ref: High (>10 years)**	1.00	1.00	1.00	1.00	1.00	1.00
Low (<10 years)	1.81 [1.36-2.40]***	1.03 [0.89-1.19]	1.11 [0.72-1.73]	2.39 [1.29-4.44]**	0.62 [0.15-2.65]	1.43 [1.02-1.99]*
Medium (=10 years)	1.11 [0.97-1.28]	1.12 [1.04-1.20]**	1.08 [0.88-1.33]	0.96 [0.68-1.34]	0.74 [0.36-1.53]	1.16 [1.03-1.32]*
**Relative income position** ^**3**^ **, Ref: > 100% of median income**	1.00	1.00	1.00	1.00	1.00	1.00
≤60% of median income	1.43 [1.208-1.72]***	1.02 [0.93-1.13]	1.87 [1.42-2.46]***	1.12 [0.74-1.68]	1.48 [0.55-3.97]	1.07 [0.88-1.29]
>60% and ≤ 100% of median income	1.18 [1.03-1.34]*	1.00 [0.93-1.07]	1.21 [1.01-1.46]*	1.13 [0.80-1.60]	1.31 [0.57-3.01]	1.00 [0.89-1.13]
**Single parenthood, Ref: No**	1.00	1.00	1.00	1.00	1.00	1.00
Yes	0.82 [0.67-0.99]*	0.89 [0.80-0.99]*	0.97 [0.74-1.28]	0.92 [0.55-1.54]	1.82 [0.32-10.38]	1.10 [0.91-1.32]
Missing	0.90 [0.59-1.38]	1.16 [0.91-1.47]	1.30 [0.69-2.42]	0.65 [0.19-2.23]	1.00 [1.00-1.00]	1.09 [0.77-1.52]
**Study region, Ref: Munich**	1.00	1.00	1.00	1.00	1.00	1.00
Leipzig	1.54 [1.27-1.86]***	1.07 [0.96-1.18]	0.84 [0.64-1.10]	1.63 [1.09-2.44]*	1.41 [0.57-3.49]	1.67 [1.45-1.91]***
Bad Honnef	0.78 [0.61-0.99]*	0.98 [0.85-1.12]	0.75 [0.51-1.11]	0.82 [0.45-1.51]	0.67 [0.12-3.61]	1.09 [0.86-1.39]
Wesel	0.67 [0.58-0.76]***	0.93 [0.86-0.99]*	0.72 [0.58-0.89]**	0.89 [0.62-1.28]	0.60 [0.27-1.33]	0.98 [0.84-1.14]
**MVPA activity time** ^**4**^ **, Ref: < 7 h/week MVPA** ^**5**^	1.00	1.00	1.00	1.00	1.00	1.00
≥7 h/week MVPA^6^	0.99 [0.88-1.12]	0.96 [0.90-1.03]	1.11 [0.93-1.32]	0.79 [0.57-1.09]	0.91 [0.42-1.98]	1.06 [0.95-1.18]

Abbreviations: BMI: body mass index; CI: confidence interval; Exp(Est): exponential function of the l’beta estimate; h: hours; %: percentage; Ref: reference category.

^1^BMI categories according to Kromeyer-Hauschild et al. 2001 [28].

^2^Maximum completed school years of either of the parents.

^3^Categorized relatively to the median equivalence income of the year 2007 (1521 EUR/month) [24].

^4^MVPA: moderate-to-vigorous physical activity (year average, dichotomized according to WHO recommendations ≥ 60 min/day MVPA) [2].

^5^WHO recommendations not met.

^6^WHO recommendations met.

***/**/*, values nominally significant at the 0.1%/1%/5% level (without adjustment for multiple testing).

Number of observations: 3356.

Model information: dependent variables: Exp(Estimate) for costs (total direct, physician, therapist, hospital, inpatient rehabilitation, parental work absence); assumptions: gamma distribution of the error terms, log-link function.

The analysis showed no statistically significant results. Different directions of estimates (extent of costs where costs occurred) were noticeable throughout cost components in the second step of the regression model: For higher MVPA (≥7 h/week) compared with lower MVPA (<7 h/week) the extent of total, physician, hospital and rehabilitation costs was 0.99fold (95% CI: 0.88-1.12), 0.96fold (95% CI: 0.90-1.03), 0.79fold (95% CI: 0.57-1.09) and 0.91fold (95% CI: 0.42-1.98), while therapist and indirect costs were 1.11fold (95% CI: 0.93-1.32) and 1.06fold (95% CI: 0.95-1.18).

Regarding further parameters of the regression model, there are single significant estimates: For a one unit increase in age, total costs are higher (1.63fold, 95% CI: 1.28-2.08). Boys showed increased total and therapist costs compared with girls. Regarding BMI, total direct costs are higher for all weight categories compared with normal weight. The highest total costs can be observed for obese children (1.89fold, 95% CI: 1.27-2.82).

More detailed analyses were done regarding the costs for further specialists as well as for further therapists. The analyses did not reveal systematic changes in comparison with the reported overall analyses on the extent of physicians and therapists costs.

### Results of the sensitivity analyses

Sensitivity analyses were conducted on all cost components and on both steps of the regression model. They did not systematically change the results in comparison with the primary analysis. In Table 5 results for sensitivity analyses of total costs are shown in comparison with the primary analysis model.

**Table 5:** **Primary and sensitivity analyses: correlation between MVPA and total direct costs**

**Type of analysis**	**Total direct costs (yes) (f.s.)**	**Total direct costs (Amount) (s.s.)**
	**Odds [95% CI]**	**Exp(Estimate) [95% CI]**
**Primary analysis model, Ref: < 7 h/week MVPA**	1.00	1.00
≥7 h/week MVPA^1^	0.95 [0.76-1.19]	0.99 [0.88-1.12]
**SAM 1: Exclusion of high utilization, Ref: < 7 h/week MVPA**	1.00	1.00
≥7 h/week MVPA^1^	0.95 [0.76-1.19]	1.05 [0.93-1.18]
**SAM 2: four-level MVPA variable, Ref: < 3.5 h/week MVPA**	1.00	1.00
≥3.5 and < 7 h/week MVPA	1.04 [0.68-1.59]	1.12 [0.89-1.41]
≥7 and < 10.5 h/week MVPA	0.92 [0.61-1.40]	0.90 [0.72-1.13]
≥10.5 h/week MVPA	1.01 [0.68-1.51]	1.18 [0.95-1.47]
**SAM 3: plus Interaction MVPA & gender, Ref: < 7 h/week MVPA**	1.00	1.00
Effect of ≥ 7 h/week MVPA if gender female	0.94 [0.69-1.28]	1.31 [1.10-1.55]**
Effect of ≥ 7 h/week MVPA if gender male	0.96 [0.69-1.32]	0.77 [0.64-0.91]**
**SAM 4: plus Interaction MVPA & BMI, Ref: < 7 h/week MVPA**	1.00	1.00
Effect of ≥ 7 h/week MVPA if normal weight	0.96 [0.76-1.23]	0.99 [0.86-1.13]
Effect of ≥ 7 h/week MVPA if severely underweight	1.21 [0.22-6.76]	2.08 [0.98-4.45]
Effect of ≥ 7 h/week MVPA if underweight	0.91 [0.36-2.29]	2.09 [1.29-3.38]**
Effect of ≥ 7 h/week MVPA if overweight, not obese	0.89 [0.31-2.54]	1.09 [0.64-1.86]
Effect of ≥ 7 h/week MVPA if obese	0.65 [0.06-6.78]	1.65 [0.71-3.83]
Effect of ≥ 7 h/week MVPA if BMI information missing	0.77 [0.26-2.29]	0.19 [0.10-0.37]***

Abbreviations: BMI: body mass index CI: confidence interval; Exp(Estimate): exponential function of l’beta estimate for costs; f.s.: first step of the model (logistic regression); h: hours; MVPA: moderate to vigorous physical activity; Odds: odds ratio estimates for costs; PA: physical activity; %: percentage; Ref: reference category; SAM: sensitivity analysis model; s.s.: second step of the model (overall generalized linear mixed regression).

***/**/*, values nominally significant at the 0.1%/1%/5% level (without adjustment for multiple testing).

Model information:

^1^effect of respectively higher MVPA (≥7 h/week) for both sexes compared with reference category lower MVPA (<7 h/week).

all SAMs were calculated using covariates of the primary analysis; number of observations vary in data sets and between steps of the regression model:

f.s.: primary analysis, SAM2, SAM3, SAM4 (N = 3356), SAM1 (N = 3355).

s.s.: primary analysis, SAM2, SAM3, SAM4 (N = 2913), SAM1 (N = 2912).

The information that can be added through sensitivity analyses is related to the second step of the regression model. In SAM 3 and SAM 4 there are some significant values: If costs occur, boys show a lower extent of total costs (0.77fold, 95%CI: 0.64-0.91), while girls show an increased extent of total costs (1.31fold, 95% CI: 1.10-1.55) for higher compared with lower MVPA. If costs occur, for underweight children total costs double for higher compared with lower MVPA.

### Recycled predictions of mean (in)direct costs and MVPA

**Figure 1:**
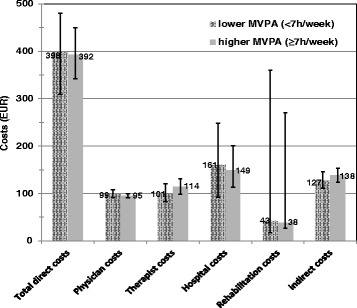
Mean (in)direct costs and MVPA (95% confidence intervals) in EUR.

Combining the two steps of the regression model, the results for the recycled predictions estimate adjusted mean costs and 95% bootstrap-percentile-intervals for both MVPA groups (shown in Figure 1). For the group fulfilling WHO recommendations (≥7 h/week) compared with the group not fulfilling WHO recommendations (<7 h/week) total direct costs accounted for 392 EUR (95% CI: 342–449 EUR) versus 398 EUR (95% CI: 309–480 EUR) and indirect costs accounted for 138 EUR (95% CI: 124–153 EUR) versus 127 EUR (95% CI: 111–146 EUR).

Examining the direct costs in more detail, for higher (≥7 h/week) compared with lower MVPA (<7 h/week) physician costs accounted for 95 EUR (95% CI: 91–100 EUR) versus 99 EUR (95% CI: 91–107 EUR), therapist costs for 114 EUR (95% CI: 98–131 EUR) versus 101 EUR (95% CI: 82–121 EUR), hospital costs for 149 EUR (95% CI: 113–201 EUR) versus 161 EUR (95% CI: 92–249 EUR) and inpatient rehabilitation costs for 38 EUR (95% CI: 26–270 EUR) versus 43 EUR (95% CI: 17–360 EUR). All effects, however, are not significant.

## Discussion

This study analyzed the association between PA, healthcare utilization and costs for children based on cross-sectional data of the 10-year follow-up from two German birth cohort studies. The results show that a majority of children fulfill WHO-recommendations of ≥ 7 h/week MVPA and seem to be quite active. No statistically significant association between PA and healthcare utilization and costs was observed. The results show different directions of association. Basically, physically active children are healthier in terms of fitness [3]. Having a better fitness, physically active children are less likely to be in need of healthcare services. But overburdening PA can also lead to physical injuries and chronic damage during child development [36]. Annually, about 17.7% of boys and 14.1% of girls (5 to 14 years old) get injured in accidents, 32.1% of these accidents happen during sports/leisure time [37]. A Dutch study shows that for the narrower age group of the 9 to 12 year-old children injury risks during leisure time might be higher in girls compared with boys [38]. In both sexes, injuries probably result in demands of healthcare services.

This study has strengths and limitations. It is the first study analyzing the association between PA, healthcare utilization and costs for children using a bottom-up approach which needs fewer assumptions regarding individual utilization compared with top-down approaches. A broad spectrum of healthcare services is captured (physician, therapist, hospital, inpatient rehabilitation costs) and as one of the first studies it even takes into account an aspect of indirect costs (parental work absence). Using an excess cost approach, the present study is able to capture all costs related to MVPA or its health consequences and to compare costs between groups of different MVPA level.

A Canadian study analyzed similar associations: Kirk et al. explored the association between health behaviors (including PA) and healthcare utilization in Canadian schoolchildren using a top-down approach and a cross-sectional study design. They linked survey data from the Children’s Lifestyle and School Performance Study (CLASS) with Nova Scotia administrative health data. To measure healthcare utilization and costs they only use physician visits and physician costs as outcome. As in the present study, Kirk et al. found no statistically significant association between PA and healthcare utilization [16], but for increasing PA, they observed a non-significant trend of increasing healthcare costs [16].

The present study is subject to some limitations. As a cross-sectional design was used, statements about causal relationships, accumulative or long-term effects of PA on healthcare utilization and costs cannot be made. In the long run, for physically active people, savings potential is assumed [39]. Rütten et al. mention an Austrian calculation that weights costs and benefits of PA resulting in an annual savings potential of circa 270 million EUR for PA. Childhood might be too early in life, to detect significant preventive effects of PA on healthcare utilization and costs, as diseases attributable to lacking PA might first occur later in life. As we focus on a narrow time frame, it seems plausible that the immediate effects of PA related injuries on healthcare utilization and costs might outweigh the preventive effects.

Preventive effects of PA on healthcare utilization and costs were assumed but even an inverse causation is conceivable. Children being less physically active might nevertheless show a higher probability of healthcare utilization and higher costs. This can be the case if children have serious (chronic) diseases that might restrain them from being physically active. The higher probability of healthcare utilization and higher costs might then not be associated with PA, but with the disease itself.

There are limitations regarding the estimation of cost data in the present study. This study was not able to account for actual expenditures, but applied updated contact prices based on mean values suggested by the AG MEG [29]. Costs can vary considerably, even within one healthcare category and particularly for hospital stays [40]. This approach and some assumptions regarding imputation methods may have caused an over- or underestimation of costs as is discussed in detail in Breitfelder et al. and Batscheider et al. [14,22]. Costs might be underestimated because of preventive effects of high education and income on costs in the study sample: Families participating in GINIplus and LISAplus have above average education levels and income compared with the German population in general [14].

The estimation of indirect costs is limited to one aspect of indirect costs (parental work absence costs) focusing on production losses in paid work. But this can only be regarded as an approximation of indirect costs because it only takes into account employed people and disregards unpaid work. Further indirect costs concerning children individually are also conceivable as for example negative effects on their education or career opportunities. Regarding utilization data, on which cost estimations are based, the study cannot exclude recall bias, because parents of participating children provide information about the previous 12 months. The authors do not assume an effect on the validity of their study [14].

As a tendency of “overreporting” PA is known from other surveys [41], an overestimation of MVPA in the present study is likely. However, PA questions of the present study were based on a questionnaire of the representative KiGGS study which was tested for overall test-retest reliability and for validity of PA questions, showing good results [42]. A further limitation regarding the accuracy of estimated MVPA arises from the fact that not the study subject himself, but his parents, report on MVPA. This method of data collection is common in studies among schoolchildren, see also KiGGS study [8].

For the construction of the exposure variable MVPA it has been assumed that the WHO recommendation of 60 minutes/day MVPA can be extrapolated to 7 h/week MVPA. This was necessary as PA in this study was recorded in h/week. It does, however, not ensure that children are physically active daily which might have an influence on possible health effects of MVPA. Further, other health behaviors like food habits may possibly confound the association between MVPA, healthcare utilization and costs and could not be taken into account.

Furthermore it has to be noted that the study sample is not representative for German children (above average education level and income background, small regional coverage, above average level of MVPA).

As in the 10-year follow-up only about 55% of the baseline individuals are included, non-response bias cannot be ruled out either.

## Conclusions

This study may be regarded as one of the first steps in investigating the association between PA, healthcare utilization and direct as well as indirect costs in children. Even if the study did not show significant results, it is important because it examined possible short-term effects in this association. Setting the focus on the association between PA and healthcare costs rather than on the association between a disease and healthcare costs, the study makes a contribution to the exploration of health behaviors and protective factors in primary prevention. Long-term effects remain to be analyzed to clarify the public health importance of PA. Therefore, further studies which apply a lifetime perspective and observe the participants from childhood into adulthood are needed. As it is scientifically known that positive health effects of PA in children are possible, the focus of further studies should be on these aspects: a better understanding of the PA types and the sport forms that generate health effects in children (paying particular attention to their growth process) and calculating its subsequent economic impact more exactly. This might strengthen and underpin the promotion of PA in children from a health economic perspective.

## Endnote

^a^We chose the reference year 2007 for reported numbers, because it was the year of data collection in the 10-year birth cohort.

## Abbreviations

AG MEG: Working Group Methods in Health Economic Evaluation
BMI: Body mass index
CI: Confidence interval
CLASS: Children’s Lifestyle and School Performance Study
DALYs: Disability-adjusted life years
ENT: Ear nose and throat specialist
et al.: et alteri/and others
EU: European Union
EUR: Euros
GINIplus: The German Infant Study on the Influence Of Nutrition Intervention plus Air Pollution and Genetics on Allergy Development
H: Hour
Inc: Incorporated
KiGGS: German Interview and Examination Survey for Children and Adolescents
LISAplus: Influence of Life-style Factors on Development of the Immune System and Allergies in East and West Germany plus Air Pollution and Genetics on Allergy Development
MVPA: Moderate-to-vigorous physical activity
NC: North Carolina
OECD: Organization for Economic Cooperation and Development
%: Percent
P: Percentile
PA: Physical activity
SAM: Sensitivity analysis model
SAS: Statistical analyzing system
USA: United States of America
vs: Versus
WHO: World Health Organization

## References

[1] Knoll M, Banzer W, Bös K (2006). Aktivität und physische Gesundheit. Handbuch Gesundheitssport.

[2] Wagner P, Brehm W (2006). Aktivität und psychische Gesundheit. Handbuch Gesundheitssport.

[3] Sygusch R, Wagner P, Opper E, Worth A (2006). Aktivität und Gesundheit im Kindes- und Jugendalter. Handbuch Gesundheitssport.

[4] Kohl HW, Craig CL, Lambert EV, Inoue S, Alkandari JR, Leetongin G (2012). The pandemic of physical inactivity: global action for public health. Lancet.

[5] World Health Organization. Global recommendations on physical activity for health. In. Geneva; 2010. 26180873

[6] World Health Organization. Global health risks: mortality and burden of disease attributable to selected major risks. In. Geneva; 2009.

[7] Pratt M, Norris J, Lobelo F, Roux L, Wang G. The cost of physical inactivity: moving into the 21st century. Br J Sports Med. 2012;(epub ahead of print):1–3. 10.1136/bjsports-2012-091810 23134760

[8] Krug S, Jekauc D, Poethko-Müller C, Woll A, Schlaud M (2012). Zum Zusammenhang zwischen körperlicher Aktivität und Gesundheit bei Kindern und Jugendlichen: Ergebnisse des Kinder- und Jugendgesundheitssurveys (KiGGS) und des Motorik-Moduls (MoMo). Bundesgesundheitsblatt Gesundheitsforschung Gesundheitsschutz.

[9] Zapf J (2006). Übergewicht als Risikofaktor und Ernährung als notwendige gesundheitsförderliche Ergänzung körperlicher Aktivierung. Handbuch Gesundheitssport.

[10] World Health Organization. Obesity and overweight. In. Geneva; 2003.

[11] Kurth BM, Schaffrath RA (2007). Die Verbreitung von Übergewicht und Adipositas bei Kindern und Jugendlichen in Deutschland: Ergebnisse des bundesweiten Kinder- und Jugendgesundheitssurveys. Bundesgesundheitsblatt Gesundheitsforschung Gesundheitsschutz.

[12] Wolfenstetter SB (2006). Adipositas und die Komorbidität Diabtetes mellitus Typ 2 bei Kindern und Jugendlichen in Deutschland: Entwicklung und Krankheitskostenanalyse. Gesundheitswesen.

[13] Wenig CM (2012). The impact of BMI on direct costs in children and adolescents: empirical findings for the German healthcare system based on the KiGGS-study. Eur J Health Econ.

[14] Breitfelder A, Wenig CM, Wolfenstetter SB, Rzehak P, Menn P, John J (2011). Relative weight-related costs of healthcare use by children - results from two German birth cohorts, GINI-plus and LISA-plus. Econ Hum Biol.

[15] Collard DC, Verhagen EA, van Mechelen W, Heymans MW, Chinapaw MJ (2011). Economic burden of physical activity-related injuries in Dutch children aged 10–12. Br J Sports Med.

[16] Kirk SF, Kuhle S, Ohinmaa A, Veugelers PJ (2012). Health behaviours and health-care utilization in Canadian schoolchildren. Public Health Nutr.

[17] Heinrich J, Brüske I, Cramer C, Hoffmann U, Schnappinger M, Schaaf B (2012). GINIplus und LISAplus: Design und ausgewählte Ergebnisse zweier deutscher Geburtskohorten zum natürlichen Verlauf atopischer Erkrankungen sowie deren Determinanten. Allergologie.

[18] Von Berg A, Krämer U, Link E, Bollrath C, Heinrich J, Brockow I (2010). Impact of early feeding on childhood eczema: development after nutritional intervention compared with the natural course - the GINIplus study up to the age of 6 years. Clin Exp Allergy.

[19] Statistisches Bundesamt: Gesundheitsberichterstattung des Bundes. Definition körperliche Aktivität. [http://www.gbe-bund.de/gbe10/abrechnung.prc_abr_test_logon?p_uid=gastg&p_aid=&p_knoten=FID&p_sprache=D&p_suchstring=10436]

[20] Centers for disease control and prevention. How much physical activity do children need? [http://www.cdc.gov/physicalactivity/everyone/guidelines/children.html]

[21] National Health Service. Physical activity guidelines for children and young people. [http://www.nhs.uk/Livewell/fitness/Pages/physical-activity-guidelines-for-young-people.aspx]

[22] Batscheider A, Zakrzewska S, Heinrich J, Teuner CM, Menn P, Bauer CP (2012). Exposure to second-hand smoke and direct healthcare costs in children - results from two German birth cohorts, GINIplus and LISAplus. BMC Health Serv Res.

[23] Deckl S (2010). Leben in Europa 2007 und 2008: Bundesergebnisse für Sozialindikatoren über Einkommen, Armut und Lebensbedingungen. Wirtschaft und Statistik.

[24] European Commission. Glossary: equivalised income. [http://ec.europa.eu/eurostat/statistics-explained/index.php/Glossary:Equivalised_disposable_income]

[25] Statistisches Bundesamt: Leben in Europa (EU-SILC). Einkommen und Lebensbedingungen in Deutschland und der Europäischen Union. [https://www.destatis.de/DE/Publikationen/Thematisch/EinkommenKonsumLebensbedingungen/LebeninEuropa/EinkommenLebensbedingungen2150300107004.pdf?__blob=publicationFile]

[26] Kleiser C, Schaffrath Rosario A, Mensink GB, Prinz-Langenohl R, Kurth BM (2009). Potential determinants of obesity among children and adolescents in Germany: results from the cross-sectional KiGGS study. BMC Public Health.

[27] Kromeyer-Hauschild K, Wabitsch M, Kunze D, Geller F, Geiß HC, Hesse V (2001). Perzentile für den Body-mass-Index für das Kinder- und Jugendalter unter Heranziehung verschiedener Stichproben. Monatsschr Kinderheilk.

[28] Kassenärztliche Bundesvereinigung. Grunddaten zur vertragsärztlichen Versorgung in Deutschland, 2008, 2010. [http://daris.kbv.de/daris.asp]

[29] Krauth C, Hessel F, Hansmeier T, Wasem J, Seitz R, Schweikert B (2005). Empirische Bewertungssätze in der gesundheitsökonomischen Evaluation - ein Vorschlag der AG Methoden der gesundheitsökonomischen Evaluation (AG MEG). Gesundheitswesen.

[30] Hodgson TA, Meiners MR (1982). Cost-of-illness methodology: a guide to current practices and procedures. Milbank Mem Fund Q Health Soc.

[31] Wacker M, Holle R, Heinrich J, Ladwig KH, Peters A, Leidl R (2013). The association of smoking status with healthcare utilization, productivity loss and resulting costs: results from the population-based KORA F4 study. BMC Health Serv Res.

[32] Ortlieb S, Schneider G, Koletzko S, Berdel D, von Berg A, Bauer CP (2013). Physical activity and its correlates in children: a cross-sectional study (the GINIplus & LISAplus studies). BMC Public Health.

[33] Dodd S, Bassi A, Bodger K, Williamson P (2006). A comparison of multivariable regression models to analyse cost data. J Eval Clin Pract.

[34] Glick HA, Doshi JA, Sonnad SS, Polsky D (2007). Economic evaluation in clinical trials.

[35] Graubard BI, Korn EL (1999). Predictive margins with survey data. Biometrics.

[36] Mellerowicz J, Matussek J, Wilke S, Leier T, Asamoah V (2000). Sportverletzungen und Schäden im Kindes- und Jugendalter - eine Übersicht. Dtsch Z Sportmed.

[37] Kahl H, Dortschy R, Ellsäßer G (2007). Verletzungen bei Kindern und Jugendlichen (1–17 Jahre) und Umsetzung von persönlichen Schutzmaßnahmen: Ergebnisse des bundesweiten Kinder- und Jugendgesundheitssurveys (KiGGS). Bundesgesundheitsblatt Gesundheitsforschung Gesundheitsschutz.

[38] Bloemers F, Collard DC, Paw MC, van Mechelen W, Twisk J, Verhagen E (2012). Physical inactivity is a risk factor for physical activity-related injuries in children. Br J Sports Med.

[39] Rütten A, Abu-Omar K, Lampert T, Ziese T (2005). Körperliche Aktivität. Gesundheitsberichterstattung des Bundes Heft 26.

[40] Woolford S, Gebremariam A, Clark SJ, Davis MM (2007). Incremental hospital charges associated with obesity as a secondary diagnosis in children. Obesity (Silver Spring, Md.).

[41] Brehm W, Bös K, Bös K, Brehm W (2006). Gesundheitssport: Ein zentrales Element der Prävention und der Gesundheitsförderung. Handbuch Gesundheitssport. 2., vollständig neu bearbeitete Auflage.

[42] Opper E, Worth A, Wagner M, Bös K (2007). Motorik-Modul (MoMo) im Rahmen des Kinder- und Jugendgesundheitssurveys (KiGGS). Bundesgesundheitsblatt Gesundheitsforschung Gesundheitsschutz.

